# Binding mode between peptidyl-tRNA hydrolase and the peptidyl-A76 moiety of the substrate

**DOI:** 10.1016/j.jbc.2025.108385

**Published:** 2025-03-04

**Authors:** Yuji Uehara, Ami Matsumoto, Tomonori Nakazawa, Akane Fukuta, Kaori Ando, Toshio Uchiumi, Natsuhisa Oka, Kosuke Ito

**Affiliations:** 1 Department of Biology, Faculty of Science, Niigata University, Niigata, Japan; 2 Department of Chemistry and Biomolecular Science, Faculty of Engineering, Gifu University, Gifu, Japan; 3 The Institute of Science and Technology, Niigata University, Niigata, Japan; 4 Institute for Glyco-core Research (iGCORE), Gifu University, Gifu, Japan; 5 Center for One Medicine Innovative Translational Research (COMIT), Gifu University, Gifu, Japan

**Keywords:** tRNA, peptidyl-tRNA hydrolase, translation, substrate recognition, X-ray crystallography, site-directed mutagenesis, enzyme kinetics

## Abstract

Peptidyl-tRNA hydrolase (Pth) hydrolyzes the ester bond between the peptide and the tRNA of peptidyl-tRNA molecules, which are the products of aborted translation, to prevent cell death by recycling tRNA. Numerous studies have attempted to elucidate the substrate recognition mechanism of Pth. However, the binding mode of the peptidyl-A76 (3′-terminal adenosine of tRNA) moiety of the substrate to Pth, especially the A76 moiety, remains unclear. Here, we present the crystal structure of *Thermus thermophilus* Pth (*Tt*Pth) in complex with adenosine 5′-monophosphate (AMP), a mimic of A76. In addition, we show the crystal structure of *Tt*Pth in which the active site cleft interacts with the C-terminal three amino acid residues of a crystallographically related neighboring *Tt*Pth molecule. Superimposition of these two crystal structures reveals that the C-terminal carboxyl group of the neighboring *Tt*Pth molecule and the 3′-hydroxyl group of AMP are located in positions favorable for ester bond formation, and we present a *Tt*Pth⋅peptidyl-A76 complex model. The complex model agrees with many previous NMR and kinetic studies, and our site-directed mutagenesis studies support its validity. Based on these facts, we conclude that the complex model properly represents the interaction between Pth and the substrate in the reaction. Furthermore, structural comparisons suggest that the substrate recognition mode is conserved among bacterial Pths. This study provides insights into the molecular mechanism of the reaction and useful information to design new drugs targeting Pth.

During protein synthesis, ribosomes often stall before reaching the stop codon due to the presence of truncated mRNA, amino acid starvation, tRNA starvation, and other phenomena (1, 2, 3). These stalled ribosomes produce peptidyl-tRNAs, which are immature translation products (1, 2, 3). The accumulation of peptidyl-tRNAs is toxic for cells because they can halt protein synthesis due to the depletion of free tRNAs (4, 5, 6). This toxic effect is solved by the activity of peptidyl-tRNA hydrolase (Pth) (7, 8, 9). Pth releases the tRNA from the peptidyl-tRNA, by cleaving the ester bond between the C-terminal end of the peptide and the 2′- or 3′-hydroxyl group of the adenosine at the 3′-end of the tRNA (A76), and makes the free tRNA available for further rounds of protein synthesis (10, 11). Pth acts on the peptidyl-tRNA released from the ribosome by the actions of ribosomal recycling factor, elongation factor G, and initiation factors (12, 13, 14, 15), as well as the peptidyl-tRNA on the ribosome that is accessible from the outside (16, 17, 18).

Pth is found in organisms belonging to all three kingdoms of life, bacteria, archaea, and eukarya and is classified into two types, Pth1 and Pth2. Pth1 is present in bacteria and eukaryotes (in mitochondria), while Pth2 exists in archaea and eukaryotes (anchored to the mitochondrial outer membrane and exposed to the cytoplasm) (10, 11, 19, 20, 21, 22). There is no significant sequence or structural similarity between Pth1 and Pth2. Pth1 adopts an α/β globular structure and functions as a monomeric enzyme, while Pth2 has a structure similar to thioredoxin and acts as a homodimeric enzyme (23, 24, 25). However, these two enzymes serve the same function of hydrolyzing the ester bond between the peptide and the tRNA of peptidyl-tRNA molecules (10, 11, 19, 20). In this report, we focus only on Pth1, and therefore, we hereafter refer to Pth1 as Pth.

To gain insight into substrate recognition by Pth at the molecular level, many structural studies of Pth in complexes with the substrate or substrate analogs have been performed. In the first crystal structure of Pth, the active site cleft was incidentally occupied by the C-terminal amino acid residues of a crystallographically related neighboring Pth molecule (23). This crystal structure provided various clues about the recognition mechanism of the peptide moiety of the substrate. An NMR chemical shift perturbation study using an RNA duplex consisting of the CCA terminus, acceptor stem, and TΨC stem was subsequently reported (26). This study identified the amino acid residues of Pth involved in the recognition of the tRNA moiety of the substrate and showed that the anticodon arm is free in the substrate recognition process. The crystal structure of Pth in complex with an RNA minihelix consisting of the CCA terminus, acceptor stem, and TΨC arm was then determined (27) and revealed the binding mode of the acceptor stem and the TΨC arm to Pth. However, in this structure, because the peptide was not attached to the tRNA construct, the 3′-terminal adenosine A76 did not bind to the active site cleft. Therefore, the binding mode between Pth and the A76 moiety has not been clarified. A small angle neutron scattering study provided a low-resolution Pth⋅peptidyl-tRNA complex model (28), which suggested the binding mode of a later step of the reaction, with little interaction between Pth and the tRNA moiety. In addition to these structural studies using macromolecules, the crystal structures of Pth in complex with small compounds (cytidine, uridine, 3′-deoxy-3′-[(*O*-methyl-L-tyrosyl)amino]adenosine, 5-aza-2′-cytidine, and cytarabine) have been determined (29, 30, 31). Furthermore, spectroscopic and thermodynamic analyses have been performed with puromycin (32). These studies provided valuable information to develop new inhibitor drugs targeting Pth. However, no predictions or discussions of the substrate binding mode, based on the binding modes of these compounds, have been reported. In addition to these experimental efforts, docking simulation studies have been performed with small compounds (3′-(*N*,*N*-diacetyl-L-lysinyl)amino-3′-deoxyadenosine, 3′-(tri-L-alanyl)adenosine, puromycin, and 1040-C) (27, 32, 33, 34), which provided valuable clues toward understanding the binding mode of the substrate. Nevertheless, there is no direct experimental evidence to demonstrate the accuracy of the results of the docking simulations. Taken together, while the recognition of the acceptor and TΨC arms of the tRNA moiety by Pth is well understood, that of the peptidyl-A76 moiety, and especially A76, remains unclear.

To reveal the binding mode between Pth and the peptidyl-A76 moiety of the substrate, we first determined the crystal structure of *Thermus thermophilus* Pth (*Tt*Pth) in complex with adenosine 5′-monophosphate (AMP), a mimic of A76 of the tRNA. In addition, we solved the crystal structure of *Tt*Pth in which the active site cleft interacts with the C-terminal amino acid residues of a crystallographically related neighboring *Tt*Pth molecule. Superimposition of these two structures revealed that the locations of the C-terminal carboxyl group of the neighboring *Tt*Pth molecule and the 3′-hydroxyl group of AMP are favorable for ester bond formation. Using these structures, we built a *Tt*Pth⋅peptidyl-A76 complex model and performed a site-directed mutagenesis study for validation. Furthermore, we discuss the conservation of the substrate recognition mode among bacterial Pths.

## Results

### Interaction between TtPth and AMP

To elucidate the binding mode of the peptidyl-A76 moiety of the substrate, we first determined the structure of *Tt*Pth in a complex with AMP by X-ray crystallography. The data collection and structure refinement statistics are summarized in Table 1. The asymmetric unit of the crystal contains two *Tt*Pth⋅AMP complexes (A and B chains) and one AMP-unbound *Tt*Pth (C chain). The structures of the two *Tt*Pth⋅AMP complexes are quite similar (root mean square deviation for equivalent Cα atoms is 0.38 Å). Thus, we present the A chain as the structure of *Tt*Pth⋅AMP throughout this paper.

**Table 1:** Data collection and refinement statistics

**Structure**	*Tt*Pth⋅AMP	*Tt*Pth⋅tripeptide
Data collection		
Beamline	PF-AR, NW12A	PF, 17A
Wavelength (Å)	1.000	0.980
Space group	*P*6_1_22	*P*6_1_22
Unit-cell parameters		
a, b, c (Å)	120.3, 120.3, 186.0	81.9, 81.9, 123.9
α, β, γ (°)	90.0, 90.0, 120.0	90.0, 90.0, 120.0
Resolution range (Å)	104.2–1.60 (1.63–1.60)^a^	70.97–2.10 (2.14–2.10)
No. of unique reflections	104,619 (5177)	15,036 (716)
Redundancy	21.2 (20.5)	18.8 (18.0)
Completeness (%)	99.9 (100.0)	100.0 (100.0)
Average I/σ(I)	83.5 (20.8)	40.2 (7.5)
*R*_merge_^b^ (%)	5.6 (21.4)	6.2 (27.7)
Refinement		
*R*_work_/*R*_free_^c^ (%)	14.4/16.8	20.1/24.8
No. of polypeptides	3	1
No. of atoms		
Protein	4320	1320
AMP	46	–
Solvent	634	95
Average B factors (Å^2^)		
Protein	19.0	39.4
AMP	15.2	–
Solvent	30.0	52.3
R.M.S. deviations		
Bond length (Å)	0.030	0.019
Bond angle (°)	2.908	2.009
Ramachandran plot		
Favored region (%)	98.4	95.8
Allowed region (%)	1.1	3.0
Outlier region (%)	0.5	1.2
PDB ID	8X5T	8X5U

a Values in parentheses are for the highest resolution shell.

b *R*_merge_ = Σ_*hkl*_Σ_*i*_ |*I*_*i*_(*hkl*) – <*I*(*hkl*)>| / Σ_*hkl*_Σ_*i*_*I*_*i*_(*hkl*), where *I*_*i*_(*hkl*) is the *i*-th intensity measurement of reflection *hkl*, including symmetry-related reflections, and <*I*(*hkl*)> is its average.

c *R*_free_ was calculated by using 5% of randomly selected reflections that were excluded from the refinement.

**Figure 1:**
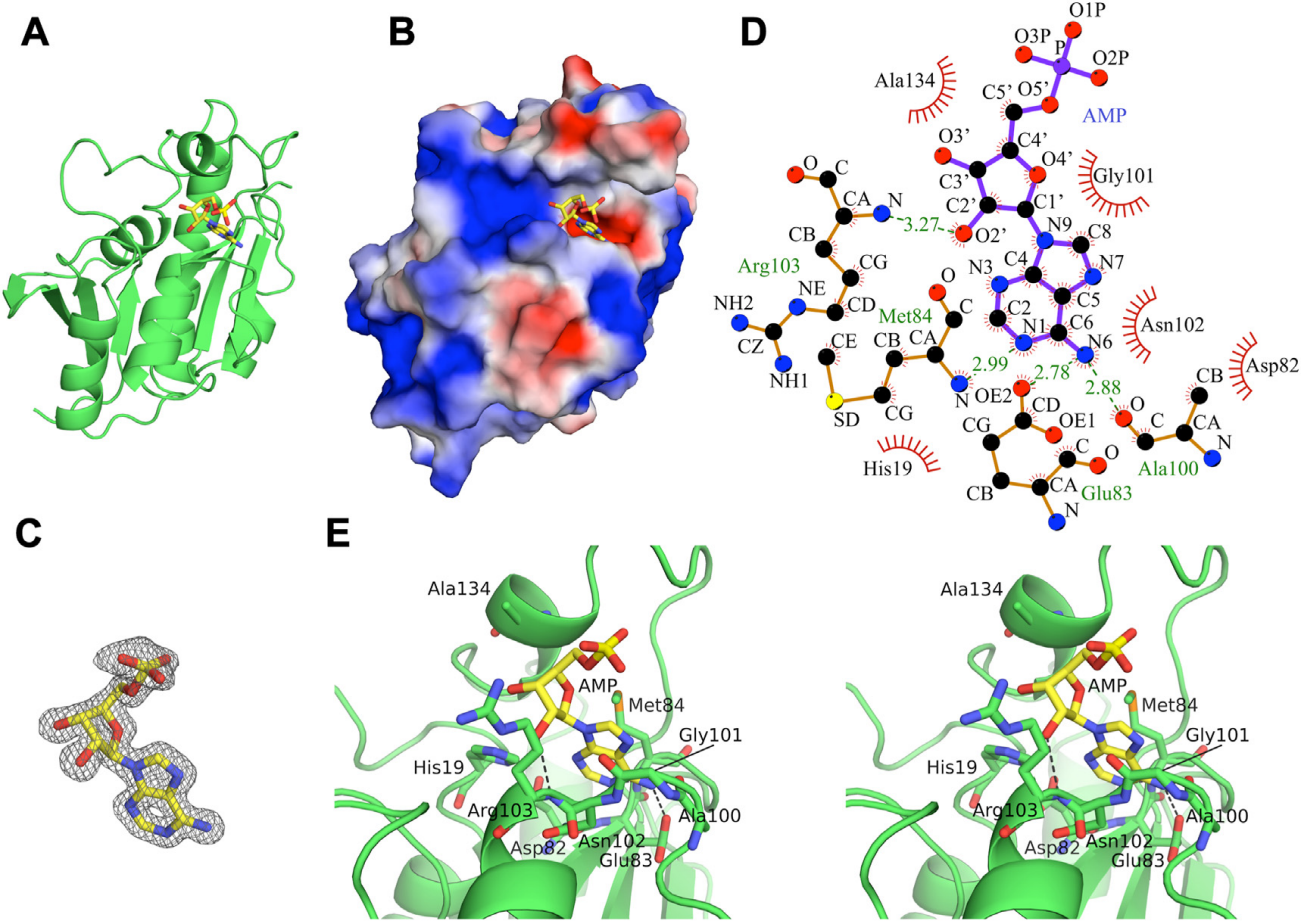
**Structure of *Tt*Pth⋅AMP**. *A* and *B*, overall structure of *Tt*Pth⋅AMP. The structure of *Tt*Pth is shown as a green cartoon model (*A*) and as a surface model colored by electrostatic potential (*red, negative; white, neutral; blue, positive*) (*B*). The bound AMP molecule is shown as a yellow stick model in both (*A* and *B*). *C*, 2*F*o − *F*c electron density maps surrounding AMP (contoured at sigma = 1.5). *D*, schematic diagram showing the interaction between *Tt*Pth and AMP. Hydrogen bonds are shown as *dashed green lines*, while van der Waals interactions are represented by *red spokes*. *E*, stereo views of the active site cleft of *Tt*Pth⋅AMP. AMP is shown as a *yellow stick* model. Amino acid residues that interact with AMP are shown as *green stick* models. Hydrogen bonds are shown as *dashed black lines*.

The structure of *Tt*Pth⋅AMP revealed that AMP is bound to an acidic pocket at the bottom of the active site cleft (Fig. 1, *A−C*). In this pocket, *Tt*Pth interacts with AMP *via* 9 amino acid residues (His19, Asp82, Glu83, Met84, Ala100, Gly101, Asn102, Arg103, and Ala134) (Fig. 1, *D* and *E*, and Table S1). Specifically, the adenine ring of AMP interacts with His19, Asp82, Glu83, Met84, Ala100, Gly101, and Asn102 *via* van der Waals interactions. Furthermore, the N6 atom of adenine forms hydrogen bonds with the side chain carboxyl oxygen of Glu83 and the main chain carbonyl oxygen of Ala100, and the N1 atom of adenine forms a hydrogen bond with the main chain amide nitrogen of Met84. The ribose moiety of AMP interacts with Met84, Gly101, Arg103, and Ala134 *via* van der Waals interactions. In addition, the hydroxyl O2′ atom of the ribose hydrogen bonds with the main chain amide nitrogen of Arg103. No interaction was observed with the phosphate moiety of AMP. These results indicate that *Tt*Pth interacts primarily with the adenine and ribose moieties of AMP, while the phosphate moiety is minimally engaged by *Tt*Pth.

To investigate the structural changes upon AMP binding, we superimposed the structure of *Tt*Pth⋅AMP with the previously determined structure of apo *Tt*Pth (PDB ID: 5ZX8) (35), although the space groups of these structures are different (Fig. S1*A*). The root mean square deviation for equivalent Cα atoms is 0.61 Å. Furthermore, we compared the distances between the crucial regions for substrate binding. The distances of the Cα atoms of the closest residues between the gate loop (Ala100) and the base loop (Asp85) in *Tt*Pth⋅AMP and apo *Tt*Pth are 7.25 Å and 7.69 Å, respectively. Likewise, the distances between the gate loop (Asn102) and the lid loop (Val137) in *Tt*Pth⋅AMP and apo *Tt*Pth are 10.39 Å and 11.03 Å, respectively. These results indicate that AMP binding does not change the structure appreciably.

### Interaction between TtPth and peptide

**Figure 2:**
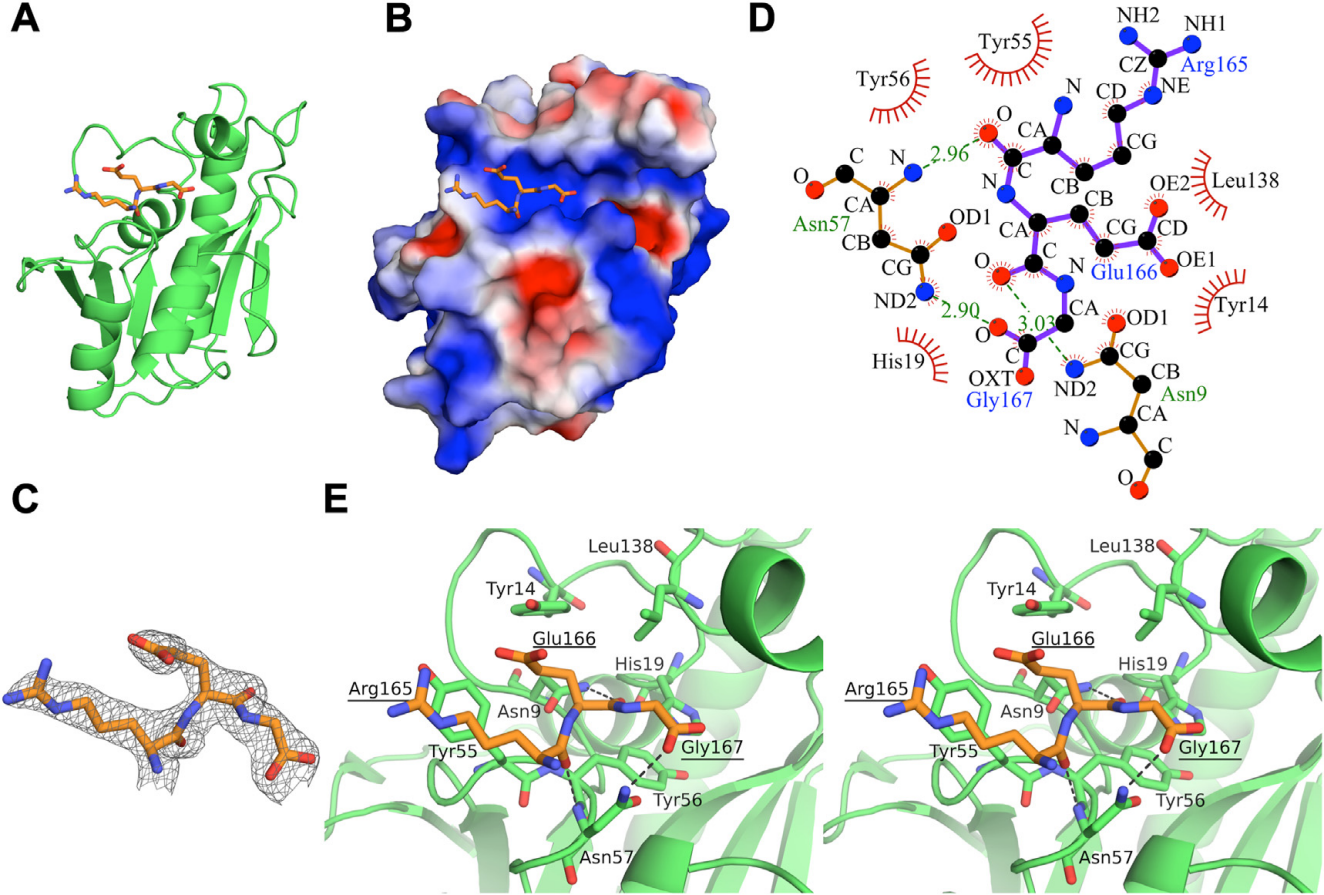
**Structure of *Tt*Pth⋅tripeptide.***A* and *B*, overall structure of *Tt*Pth⋅tripeptide. The structure of *Tt*Pth is shown as a *green cartoon* model (*A*) and as a surface model colored by electrostatic potential (*red, negative; white, neutral; blue, positive*) (*B*). The tripeptide is shown as an *orange stick* model in both (*A* and *B*). *C*, 2*F*o − *F*c electron density maps surrounding the tripeptide (contoured at sigma = 1.4). *D*, schematic diagram showing the interaction between *Tt*Pth and the tripeptide. Hydrogen bonds are shown as *dashed green lines*, while van der Waals interactions are represented by *red* spokes. *E*, stereo views of the active site cleft of *Tt*Pth. The tripeptide is shown as an *orange stick* model. Amino acid residues that interact with the tripeptide are shown as *green stick models*. Hydrogen bonds are shown as *dashed black lines*. The amino acid numbers of the tripeptide are underlined.

To elucidate the binding mode of the peptidyl-A76 moiety of the substrate, we next attempted to crystallize *Tt*Pth with 3′-(*N*-acetyl-L-alanyl)amino-3′-deoxyadenosine, a small nonhydrolyzable substrate analog that includes A76, the first amino acid, and the amide bond that mimics the first peptide bond (Fig. S2*A*). We tried many crystallization conditions using sparse matrix kits and obtained several crystals. However, the electron density of the substrate analog was not observed. In all of the crystals, the active site region contacts the C-terminal α-helix of a crystallographically-related neighboring *Tt*Pth molecule. This contact may have caused problems in the substrate analog binding. Thus, we next used a C-terminal α-helix (C-terminal 16 amino acid residues) deletion mutant and performed crystallization trials. Unfortunately, we could not obtain the desired crystals of *Tt*Pth in complex with the substrate analog. Instead, we obtained crystals of *Tt*Pth in which the C-terminal three amino acid residues (Arg165-Glu166-Gly167) of a crystallographically-related neighboring *Tt*Pth molecule penetrate and interact with the active site cleft (Figs. 2, *A*−*C* and S2*B*). Hereafter, we refer to this structure as *Tt*Pth⋅tripeptide. The data collection and structure refinement statistics are summarized in Table 1.

The *Tt*Pth⋅tripeptide structure showed that the tripeptide, Arg165-Glu166-Gly167, interacts with 7 amino acid residues in the active site cleft (Asn9, Tyr14, His19, Try55, Tyr56, Asn57, and Leu138) (Fig. 2, *D* and *E*, and Table S2). Specifically, for Arg165 of the tripeptide, the main chain interacts with Try55, Tyr56, and Asn57, and the side chain interacts with Tyr55 *via* van der Waals interactions. In addition, the main chain carbonyl oxygen of Arg165 forms a hydrogen bond with the main chain amide nitrogen of Asn57. For Glu166, the main chain interacts with Asn9, His19, Tyr56, and Leu138, and the side chain interacts with Asn9, Tyr14, and Tyr55 *via* van der Waals interactions. Furthermore, the main chain carbonyl oxygen of Glu166 forms a hydrogen bond with the side chain amide nitrogen of Asn9. For Gly167, the main chain interacts with Asn57 *via* a van der Waals interaction. In addition, one of the C-terminal carboxyl oxygens of Gly167 forms a hydrogen bond with the side chain amide nitrogen of Asn57.

To investigate the structural changes upon tripeptide binding, we superimposed the structures of *Tt*Pth⋅tripeptide with the previously determined structure of apo *Tt*Pth (PDB ID: 5ZX8) (35), although the space groups of these structures are different (Fig. S1*B*). The root mean square deviation for equivalent Cα atoms is 0.80 Å, indicating that the overall structure of *Tt*Pth does not significantly change by the peptide binding. In terms of the crucial regions for the substrate binding, the Cα atom distances of the closest residues between the gate loop (Ala100) and the base loop (Asp85) in *Tt*Pth⋅tripeptide and apo *Tt*Pth are 9.26 Å and 7.69 Å, respectively. Likewise, the distances between the gate loop (Asn102) and the lid loop (Val137) in *Tt*Pth⋅tripeptide and apo *Tt*Pth are 11.71 Å and 11.03 Å, respectively. The cleft between the base loop and the gate loop seems to open upon peptide binding. However, the structure around Ala100 of *Tt*Pth⋅tripeptide is somewhat ambiguous, due to poor electron density. Further experiments are needed to elucidate the motion of the base loop and the gate loop upon peptide binding.

### Prediction of the binding mode between TtPth and the peptidyl-A76 moiety of the substrate

To examine the positional relationship between the AMP and the tripeptide, we superimposed the structures of *Tt*Pth⋅AMP and *Tt*Pth⋅tripeptide. The result showed that the locations of the 3′-OH group of the AMP and the C-terminal carboxyl group of the tripeptide are close to each other (the distance between the O3′ atom of AMP and one of the carboxyl oxygen atoms of the tripeptide is 0.86 Å) and almost favorable for ester bond formation (Fig. S3*A*). Furthermore, the 3′-OH group of the AMP and the C-terminal carboxyl group of the tripeptide are located in the vicinity of the catalytic center residue His19 (Fig. S3*A*). Based on these facts, we infer that the structures of *Tt*Pth⋅AMP and *Tt*Pth⋅tripeptide both represent the interaction between *Tt*Pth and a part of the substrate.

**Figure 3:**
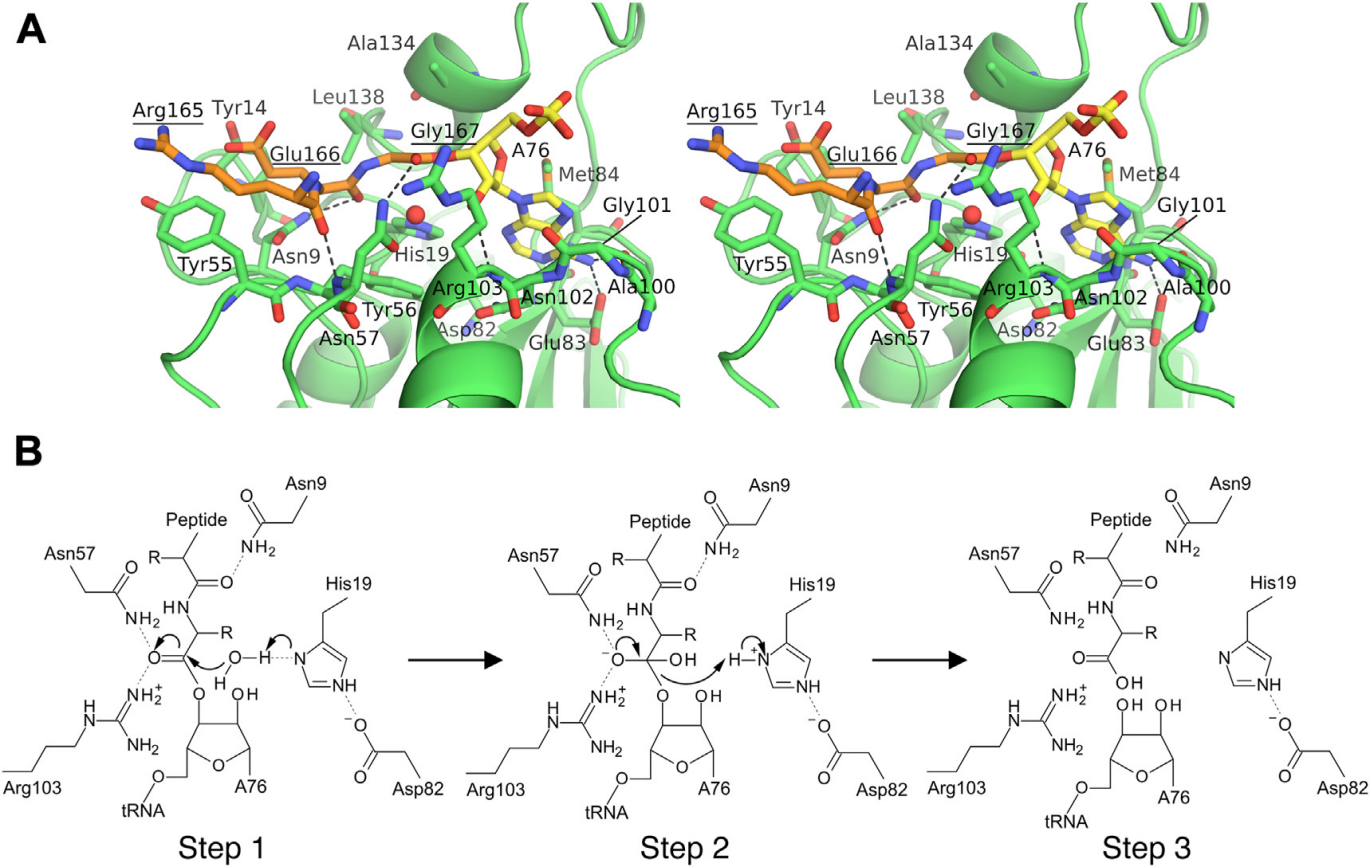
**Structure of the *Tt*Pth⋅peptidyl-A76 complex model, and reaction model of the peptidyl-tRNA hydrolysis.***A*, stereo view of the *Tt*Pth⋅peptidyl-A76 complex model. The peptide and A76 moieties of peptidyl-A76 are shown as *orange* and *yellow stick models*, respectively. Amino acid residues that interact with peptidyl-A76 are shown as *green stick models*. Hydrogen bonds are shown as *dashed black lines*. The water molecule near His19, which nucleophilically attacks the ester carbon, is represented by a *red* sphere. The amino acid numbers of the peptide moiety of the substrate are underlined. *B*, schematic representation of the catalytic mechanism of peptidyl-tRNA hydrolysis.

To gain further insights into the binding mode of the peptidyl-A76 moiety, we next tried to build a *Tt*Pth⋅peptidyl-A76 complex model. As described above, the superimposition of the structures of *Tt*Pth⋅AMP and *Tt*Pth⋅tripeptide showed that an ester bond is possible between the 3′-OH group of the AMP and the C-terminal carboxyl group of the tripeptide (Fig. S3*A*). Thus, we built the peptidyl-A76 part of the complex model by connecting these groups, while considering the ester bond stereochemistry (Fig. 3*A* and PDB S1). Regarding the *Tt*Pth part of the complex model, we utilized the coordinates of *Tt*Pth from *Tt*Pth⋅AMP without structural modifications. However, because the structures of *Tt*Pth in both *Tt*Pth⋅AMP and *Tt*Pth⋅tripeptide are quite similar (root mean square deviation for equivalent Cα atoms is 0.80 Å), the interactions between *Tt*Pth and the tripeptide observed in *Tt*Pth⋅tripeptide are almost completely preserved in the complex model (Fig. 3*A* and PDB S1).

Previously, we determined the structure of *Escherichia coli* Pth (*Ec*Pth) in complex with the CCA-acceptor-TΨC domain of tRNA (27). In this structure, the CCA terminus did not interact with the active site cleft, and thus the binding mode of the 3′-terminal adenosine A76 of tRNA to the enzyme remained unclear. Interestingly, the superimposition of the structures of the *Tt*Pth⋅peptidyl-A76 complex model and the *Ec*Pth⋅CCA-acceptor-TΨC domain complex showed that the 3′-terminal adenosine A76 of the CCA-acceptor-TΨC domain can reach and overlap with the A76 part of the *Tt*Pth⋅peptidyl-A76 complex model by a conformational change of the CCA terminal backbone (Fig. S3*B*). This finding supports the validity of the *Tt*Pth⋅peptidyl-A76 complex model.

Considering our present complex model, *Tt*Pth is predicted to interact with *N*-acetyl-aminoacyl-tRNA, the shortest substrate of Pth, *via* Asn9, His19, Tyr56, Asn57, Asp82, Glu83, Met84, Ala100, Gly101, Asn102, Arg103, Ala134, and Leu138 in the active site cleft (Fig. 3*A* and PDB S1). Among these residues, Asn9, His19, Asn57, Glu83, Met84, and Leu138 are conserved between *Tt*Pth and *Ec*Pth (Fig. S4) and predicted to interact with *N*-acetyl-aminoacyl-tRNA *via* their side chains (Fig. 3*A* and PDB S1). Previous site-directed mutagenesis studies using *Ec*Pth and diacetyl-lysyl-tRNA^Lys^, one of the shortest substrates, demonstrated that the replacement of amino acid residues corresponding to Asn9, His19, Asn57, and Met84 of *Tt*Pth with alanine dramatically reduced the *k*_cat_/*K*_m_ values (23, 26, 36, 37). In addition, NMR chemical shift perturbation studies showed that the spectra of these amino acid residues were perturbed upon the addition of substrate analogs, indicating their involvement in substrate binding (26, 33). These facts provide further support for the validity of the *Tt*Pth⋅peptidyl-A76 complex model.

Among the amino acid residues that are predicted to interact with *N*-acetyl-aminoacyl-tRNA *via* their side chains in the active site cleft, site-directed mutagenesis studies for Glu83, Asn102, Arg103, and Leu138 have not been performed. We, therefore, replaced these amino acid residues of *Tt*Pth with alanine and measured the kinetic parameters, using diacetyl-lysyl-tRNA^Lys^ as the substrate. The replacements of Glu83, Asn102, and Arg103 with alanine markedly reduced the *k*_cat_/*K*_m_ values (Table 2), supporting our complex model, while the replacement of Leu138 had little effect (Table 2). Leu138 may contribute to the substrate recognition by hydrophobic interactions, and the substituted alanine could play a similar role. These interactions may be the reason why hydrophobic amino acids are conserved at this position (Fig. S4).

**Table 2:** Kinetic parameters of the *Tt*Pth variants

*Tt*Pth variants	*k*_cat_ (s^−1^)	*K*_m_ (μM)	Relative *k*_cat_/*K*_m_
WT	0.90 ± 0.06	2.3 ± 0.8	100
E83A	0.024 ± 0.002	3.5 ± 1.1	1.8
N102A	0.029 ± 0.005	5.3 ± 2.0	1.3
R103A	0.12 ± 0.01	4.9 ± 1.4	6.1
L138A	0.70 ± 0.04	3.2 ± 1.7	55.2

Data are expressed as mean ± SD (n = 3).

### Comparison of the binding modes of peptidyl-A76 and small inhibitor compounds

The following structures of Pth in complex with small inhibitor compounds were previously determined: *Acinetobacter baumannii* Pths in complex with cytidine, uridine, or cytarabine, and *Pseudomonas aeruginosa* Pths in complex with 5-aza-2′-cytidine or 3′-deoxy-3′-[(*O*-methyl-L-tyrosyl)amino]adenosine (29, 30, 31). To compare the binding modes of these inhibitors with that of peptidyl-A76, we performed structural superimpositions. The results showed that cytidine and uridine are bound to the position where the peptide moiety of peptidyl-A76 is bound (Fig. S5, *A* and *B*). Cytarabine is not bound to the active site cleft; therefore, cytarabine and peptidyl-A76 do not superimpose at all (Fig. S5*C*). The binding positions of 5-aza-2′-cytidine and 3′-deoxy-3′-[(*O*-methyl-L-tyrosyl)amino]adenosine are similar to that of peptidyl-A76 (Fig. S5, *D* and *E*). However, the base rings of 5-aza-2′-cytidine and 3′-deoxy-3′-[(*O*-methyl-L-tyrosyl)amino]adenosine do not deeply penetrate the active site cleft, like the case of the adenine ring of peptidyl-A76 (Fig. S5, *D* and *E*). In addition, the adenine ring of 3′-deoxy-3′-[(*O*-methyl-L-tyrosyl)amino]adenosine adopts the syn-conformation, whereas the adenine ring of peptidyl-A76 adopts the anti-conformation (Fig. S5*E*).

## Discussion

In the present *Tt*Pth⋅peptidyl-A76 complex model, the main chains of the peptide moiety of peptidyl-A76 bind at the bottom of the active site cleft, and the side chains of the peptide moiety point toward the outside of the enzyme (Fig. 3*A* and PDB S1). This binding mode is consistent with the fact that the enzyme recognizes the peptide moiety of the substrate in a sequence-independent manner. Moreover, the present *Tt*Pth⋅peptidyl-A76 complex model is also compatible with the fact that the enzyme only recognizes the peptidyl-tRNA, but not the aminoacyl-tRNA (10, 11). Specifically, Asn9, the strictly conserved residue responsible for discrimination between peptidyl- and aminoacyl-tRNAs (Fig. S4) (33), forms a hydrogen bond with the carbonyl oxygen of the first peptide bond from the C-terminus (peptide bond between Glu166 and Gly167) (Fig. 3*A* and PDB S1). This hydrogen bond cannot be formed with aminoacyl-tRNAs.

To date, the hydrolysis reaction mechanism of Pth has been proposed as follows (27, 37, 38) (Fig. 3*B*): Step 1) A conserved active site asparagine residue (Asp82 in *Tt*Pth) stabilizes the fully protonated form of a conserved active site histidine (His19 in *Tt*Pth), allowing the latter to accept a proton from a water molecule. This water molecule then nucleophilically attacks the ester carbon of the peptidyl-tRNA. Step 2) A tetrahedral intermediate is formed and stabilized by the formation of a hydrogen bond with a conserved active site asparagine (Asn57 in *Tt*Pth). Step 3) The tetrahedral intermediate decomposes, producing the peptide and the tRNA. The present *Tt*Pth⋅peptidyl-A76 complex model strongly supports this proposed reaction mechanism for the following reasons. First, in the present model, the ester bond of the peptidyl-tRNA exists in the vicinity of His19 (Fig. 3*A* and PDB S1). Furthermore, a water molecule that nucleophilically attacks the ester carbon can be placed in a position to form a hydrogen bond with His19 (Fig. 3*A* and PDB S1). Second, in the present model, the ester carbonyl oxygen, which will be the oxyanion in the intermediate state, forms a hydrogen bond with the side chain amide group of Asn57 (Fig. 3*A* and PDB S1). Therefore, the tetrahedral intermediate is stabilized.

A previous study of *E. coli* Pth (*Ec*Pth) proposed that two asparagine residues, Asn68 and Asn114, contribute to stabilizing the tetrahedral intermediate by forming hydrogen bonds with its oxyanion (27) (Fig. S6*A*). Of these two asparagine resides, Asn68 is conserved as Asn57 in *Tt*Pth (Fig. S4) and can form a hydrogen bond with the oxyanion of the intermediate in the present *Tt*Pth⋅peptidyl-A76 complex model, as described above (Fig. 3*A* and PDB S1). Meanwhile, Asn114 in *Ec*Pth is not conserved in *Tt*Pth and changed to Arg103 in *Tt*Pth (Fig. S4). However, Arg103 of *Tt*Pth, similar to Asn114 of *Ec*Pth, is in a position to be able to interact with the oxyanion of the tetrahedral intermediate (Fig. 3*A* and PDB S1). In addition, the replacement of Arg103 of *Tt*Pth with alanine markedly reduced the *k*_cat_/*K*_m_ value (Table 2). Thus, we infer that Arg103 of *Tt*Pth, like Asn114 of *Ec*Pth, also contributes to stabilizing the tetrahedral intermediate (Fig. S6, *A* and *B*). Interestingly, positively charged amino acid residues are also found at the sequence position equivalent to Arg103 of *Tt*Pth in some Pths from thermophilic bacteria (Fig. S7). These alterations may be necessary to exert the activity at high temperatures.

**Figure 4:**
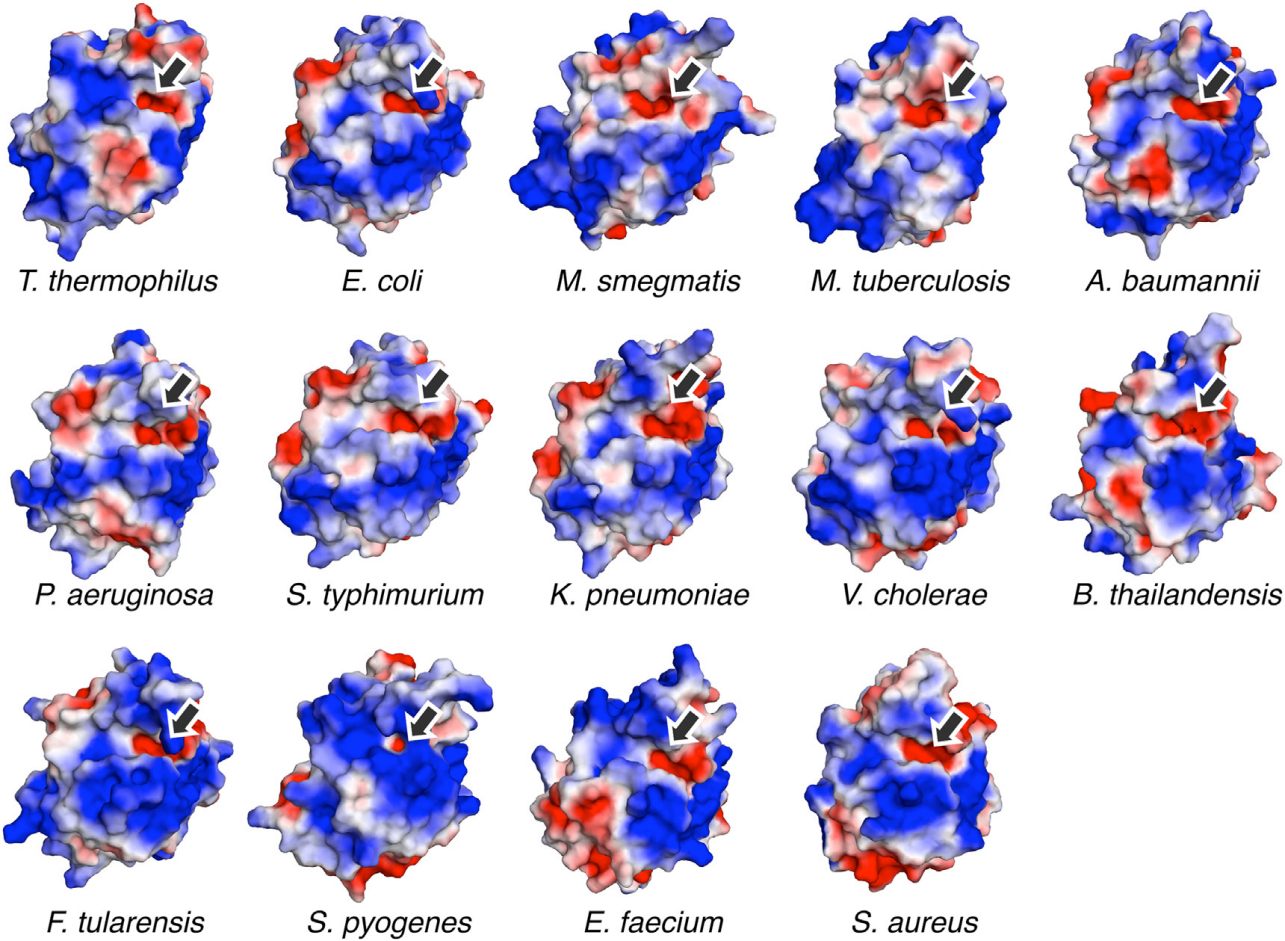
**Conservation of the acidic pockets at the bottom of the active site clefts**. Structure-determined Pths are shown by electrostatic surface models (*red, negative; white, neutral; blue, positive*). Pths from *Thermus thermophilus* (PDB ID: 8X5T) (this study), *Escherichia coli* (PDB ID: 2PTH) (23), *Mycobacterium smegmatis* (PDB ID: 3KJZ) (57), *Mycobacterium tuberculosis* (PDB ID: 2Z2I) (58), *Acinetobacter baumannii* (PDB ID: 4JY7) (29), *Pseudomonas aeruginosa* (PDB ID: 4FYJ) (59), *Salmonella typhimurium* (PDB ID: 4P7B) (60), *Klebsiella pneumoniae* (PDB ID: 7BRD) (61), *Vibrio cholerae* (PDB ID: 4ZXP) (38), *Burkholderia thailandensis* (PDB ID: 3V2I) (62), *Francisella tularensis* (PDB ID: 3NEA) (63), *Staphylococcus pyogenes* (PDB ID: 4QT4) (64), *Enterococcus faecium* (PDB ID: 7Y52) (34), and *Staphylococcus aureus* (PDB ID: 4YLY) (65). Arrows indicate conserved acidic pockets at the *bottom* of the active site clefts, which putatively accommodate the adenine ring of A76 of the tRNA.

**Figure 5:**
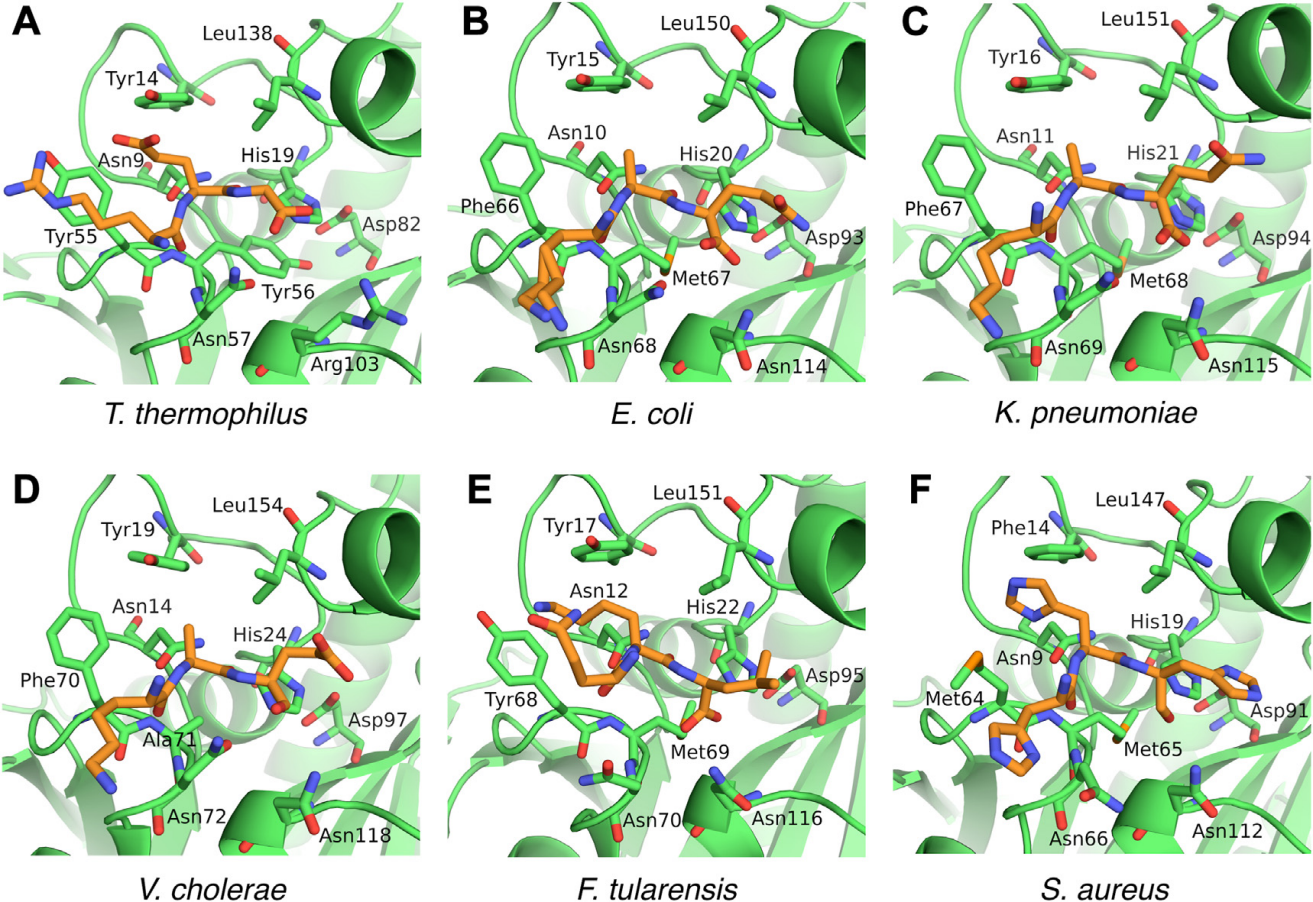
**Comparison of the Pth and peptide interaction modes**. Pths in which the C-terminus of a crystallographically-related neighboring Pth molecule is bound to the active site cleft were selected from the structure-determined Pths. Pths from (*A*) *Thermus thermophilus* (PDB ID: 8X5U) (this study), (*B*) *Escherichia coli* (PDB ID: 2PTH) (23), (C) *Klebsiella pneumoniae* (PDB ID: 7BRD) (61), (*D*) *Vibrio cholerae* (PDB ID: 5ZK0) (66), (*E*) *Francisella tularensis* (PDB ID: 3NEA) (63), and (*F*) *Staphylococcus aureus* (PDB ID: 4YLY) (65). The C-terminal amino acid residues of crystallographically related neighboring molecules are shown as *orange stick models*. Amino acid residues that interact with the C-terminal amino acid residues are shown as *green stick models*. In (*D* and *F*), C-terminal carboxyl oxygen atoms are not shown because the coordinates of these atoms are missing in the PDB coordinate files. Note that the Pth from *V. cholerae* shown in (*D*) is the M71A mutant. This 71st amino acid residue is originally methionine.

In this research, the structure of the *Tt*Pth⋅AMP complex revealed that the adenine ring of the A76-mimicking AMP binds to the acidic pocket in the active site cleft. This acidic pocket is also found in all Pth structures determined to date (Fig. 4). The side chains of Asp82, Glu83, and Asp85 are the main contributors to the negative charge of this pocket, and these residues are highly conserved across other Pths (Figs. S4 and S7). Both glutamic acid and aspartic acid are found at position 83, but this variation presumably does not disrupt the nature of the pocket. The crystal structure of *Tt*Pth⋅tripeptide showed that the C-terminal amino acid residues of a crystallographically-related neighboring molecule bind to the active site cleft, in a manner mimicking a peptide moiety of the substrate. A similar interaction is also found in several crystal structures of Pths from other species (Fig. 5). Furthermore, the key catalytic residues involved in the hydrolysis reaction (His19, Asn57, and Asp82 in *Tt*Pth) are strictly conserved and located in equivalent positions (Figs. 5 and S4). The other key catalytic residue, which corresponds to Arg103 of *Tt*Pth, is also conserved as asparagine or a positively charged amino acid residue, and exists in an equivalent position (Figs. 5, S4, and S7). These facts indicate that the substrate binding mode and the hydrolysis reaction mechanism of Pth are highly conserved beyond species.

## Experimental procedures

### Plasmid construction

Plasmids for the expression of the C-terminal 16 amino acid deletion mutant and site-directed mutants of *T. thermophilus* HB8 Pth (*Tt*Pth) were prepared by PCR, using a plasmid carrying the wild type *Tt*Pth gene as the template (39). The PCR primers are listed in Table S3. All resulting plasmids include an N-terminal His-tag and a thrombin cleavage site.

### Protein expression and purification

The C-terminal 16-amino acid deletion mutant of *Tt*Pth (ΔC16 *Tt*Pth) was expressed in *E. coli* strain BL21(DE3) at 37 °C in LB medium containing 15 μg/ml kanamycin. The protein expression was induced by adding 0.5 mM isopropyl-*β*-D-thiogalactopyranoside (IPTG) to an early exponential phase culture (OD_600_ ∼ 0.6) and continuing the culture for 3 h at 37 °C. The cells were harvested by centrifugation, suspended in buffer A (20 mM Hepes-KOH, pH 7.6, 150 mM KCl, and 7 mM *β*-mercaptoethanol), and then disrupted by sonication. After centrifugation, the supernatant was loaded onto a Ni-chelating column containing cOmplete His-Tag Purification Resin (Sigma-Aldrich), equilibrated with buffer B (20 mM Hepes-KOH, pH 7.6, 1 M NH_4_Cl, 5% (v/v) glycerol, and 7 mM *β*-mercaptoethanol). After washing the column with buffer B containing 20 mM imidazole-HCl, pH 8.0, the protein was eluted with buffer B containing 250 mM imidazole-HCl, pH 8.0. The fractions containing ΔC16 *Tt*Pth were pooled and dialyzed against buffer B. To digest the N-terminal His-tag, thrombin (GE Healthcare) was added to the solution in a ratio of 6.25 U of thrombin to 1 mg of ΔC16 *Tt*Pth, and the sample solution was incubated at 22 °C for ∼16 h. Next, to remove the isolated His-tag and the undigested His-tagged ΔC16 *Tt*Pth, the protein solution was loaded onto the Ni-chelating column equilibrated with buffer B. The flow-through fraction was collected, dialyzed against buffer A, and concentrated using a Vivaspin centrifugal concentrator (3 kDa cutoff size, Sartorius). For further purification, the sample was then applied to a HiLoad 26/60 Superdex 75 pg column (GE Healthcare) equilibrated with buffer A. The purified sample was dialyzed against buffer C (10 mM Hepes-KOH, pH 7.6, and 7 mM *β*-mercaptoethanol), concentrated, and stored at −80 °C prior to use.

The wild-type and site-directed mutants of *Tt*Pth used in enzymatic analyses were expressed, harvested, and lysed in the same manner as ΔC16 *Tt*Pth. Next, the cell supernatants were incubated at 70 °C for 15 min to denature the *E. coli* proteins, and the cell debris was removed by centrifugation. The subsequent His-tag affinity purification and His-tag removal were performed for ΔC16 *Tt*Pth. The purified proteins were dialyzed against buffer C, concentrated, and stored at −80 °C prior to use. The expression and purification of wild-type *Tt*Pth used in crystallization were performed as described previously (39).

### Chemical synthesis of 3′-(N-acetyl-L-alanyl)amino-3′-deoxyadenosine

The detailed methods of the chemical synthesis of 3′-(*N*-acetyl-L-alanyl)amino-3′-deoxyadenosine are described in the Supporting Experimental Procedures. Briefly, 3′-amino-5′-*O*-(*tert*-butyldiphenylsilyl)-3′-deoxyadenosine (40) was condensed with *N*-Fmoc-L-alanine using DMT-MM at the 3′-position. Subsequent removal of the Fmoc group by DBU afforded 3′-(L-alanyl)amino-5′-*O*-TBDPS-3′-deoxyadenosine (29% yield, 2 steps). The product was converted into 3′-(*N*-acetyl-L-alanyl)amino-5′-*O*-TBDPS-3′-deoxyadenosine by DMT-MM-mediated condensation with acetic acid. Finally, 3′-(*N*-acetyl-L-alanyl)amino-3′-deoxyadenosine was obtained by 5′-TBDPS group removal with NH_4_F and purification by reversed-phase column chromatography (20% overall yield, 4 steps). The structure of the compound was confirmed by NMR spectroscopy (^1^H and ^13^C NMR) and high-resolution mass spectrometry (ESI-TOF, *m*/*z* [M + H]^+^ calcd. for C_15_H_22_N_7_O_5_^+^ 380.1677; found 380.1687.

### Crystallization

Crystals of *Tt*Pth⋅AMP were obtained from a drop made by mixing 1 μl of a solution containing 20 mg/ml wild-type *Tt*Pth in buffer C and an equal volume of reservoir solution, containing 0.9 M K/Na tartrate, 0.1 M MES buffer, pH 5.8, and 40 mM adenosine 5′-monophosphate. The drop was equilibrated over 450 μl of the reservoir solution by the sitting drop vapor diffusion method at 20 °C. The crystals grew to full size (80 × 80 × 200 μm^3^) within 3 days.

Crystals of *Tt*Pth⋅tripeptide were obtained from a drop composed of 1 μl of a solution containing 13 mg/ml ΔC16 *Tt*Pth and 20 mM 3′-(*N*-acetyl-L-alanyl)amino-3′-deoxyadenosine in buffer C and an equal volume of reservoir solution, containing 0.5 M (NH_4_)_2_SO_4_, 0.1 M Tris-HCl, pH 7.5, 30% (v/v) PEG600, and 10% (v/v) glycerol. The drop was equilibrated over 450 μl of the reservoir solution by the sitting drop vapor diffusion method at 20 °C. The crystals grew to full size (150 × 150 × 300 μm^3^) within 3 days.

### Data collection and structure determination

Prior to data collection, crystals of *Tt*Pth⋅AMP were soaked in the reservoir solution containing 25% (v/v) glycerol as a cryoprotectant. For the crystals of *Tt*Pth⋅tripeptide, an additional cryoprotectant was not needed to prevent ice-ring formation. Diffraction data of *Tt*Pth⋅AMP were collected with a wavelength of 1.000 Å at 95 K on beamline NW12A of the Photon Factory Advanced Ring (PF-AR; Tsukuba, Japan), using an ADSC Quantum 210 CCD detector. Diffraction data of *Tt*Pth⋅tripeptide were collected with a wavelength of 0.980 Å at 95 K on beamline 17A of the Photon Factory (PF; Tsukuba, Japan), using a Dectris Pilatus3 S6M PAD detector. The indexing, integration, and scaling of the diffraction data were performed with the program HKL-2000 (41).

The initial structures of both *Tt*Pth⋅AMP and *Tt*Pth⋅tripeptide were obtained by the molecular replacement method with the program MOLREP (42), from the CCP4 program suite (43). The structure of apo-form *Tt*Pth (PDB ID: 5ZX8) (35) was used as the search model. The solutions were then improved by iterative cycles of manual model building with the program COOT (44) and maximum likelihood reﬁnement with the program REFMAC5 (45). The 2*F*o − *F*c and *F*o − *F*c electron density maps were used as the model building references and 5% of the reflections were used to calculate the *R*_free_ values. After the model improvement for the polypeptide chains, small-molecule compounds were introduced into the models. Next, water molecules were added using the programs ARP/wARP (46) and COOT, with criteria in which the electron density peaks above both 1.0 sigma in the 2*F*o − *F*c map and 3.4 sigma in the *F*o − *F*c map were used.

### Structure and sequence analyses

The quality of the model was checked using the programs COOT and PROCHECK (47). The calculations of the root mean square deviations between pairs of equivalent Cα atoms were executed using the program SUPERPOSE (48). Secondary structures were assigned using the program DSSP (49). The surface electrostatic potentials were calculated using the program APBS (50). The detection and the figure preparation of protein-ligand interactions were performed using the program LIGPLOT (51). Structure figures were prepared using the program PyMOL (https://www.pymol.org/). Multiple sequence alignments were performed using the program ClustalW (52) and the figures were produced using the program ESPript (53).

### Preparation of diacetyl-Lys-tRNA^Lys^

*E. coli* tRNA^Lys^ was synthesized by *in vitro* transcription. The template DNA for *in vitro* transcription was obtained by PCR, using a plasmid carrying the *E. coli* tRNA^Lys^(CUU) gene as the template (54). The PCR primers are listed in Table S3. The *in vitro* transcription and the purification of the RNA sample were performed as described previously (27, 55). Purified tRNA^Lys^ was aminoacylated at 37 °C in 50 mM HEPES-KOH (pH 7.6), containing 20 mM KCl, 10 mM MgCl_2_, 4 mM ATP, 7 mM *β*-mercaptoethanol, 0.01% BSA, 10 μM tRNA^Lys^, 20 μM L-[^14^C]lysine (280 Ci/mol), and 150 nM purified *E. coli* Lysyl-tRNA synthetase (56). After a 30-min incubation, the aminoacylated sample was extracted with phenol and chloroform and then precipitated with 2-propanol in the presence of 0.3 M sodium acetate. Subsequently, the acetylation reaction was performed as described previously (27). The final product, diacetyl-[^14^C]Lys-tRNA^Lys^, was dissolved in 5 mM sodium acetate buffer (pH 5.5) and stored at −80 °C.

### Peptidyl-tRNA hydrolase assay

The peptidyl-tRNA hydrolase assay was performed as described previously (27), using 3.5 to 20 μM diacetyl-[^14^C]Lys-tRNA^Lys^ and catalytic amounts of *Tt*Pth variants (5–200 nM).

## Data availability

The coordinates and structure factors have been deposited in the RCSB Protein Data Bank (https://www.rcsb.org) with the PDB IDs 8X5T for *Tt*Pth⋅AMP and 8X5U for *Tt*Pth⋅tripeptide.

## Supporting information

This article contains supporting information (27, 29, 30, 31, 35, 40).

## Conﬂict of interest

The authors declare that they have no conﬂicts of interest with the contents of this article.

## Supporting information

Supporting_PDB_S1

Uehara_et_al_JBC_SI
