# Supplementary material for: Binding mode between peptidyl-tRNA hydrolase and the peptidyl-A76 moiety of the substrate
Source: J Biol Chem. 2025 Mar 4;301(4):108385. doi: 10.1016/j.jbc.2025.108385 (PMC11994314; doi:10.1016/j.jbc.2025.108385)
Supplement: Uehara_et_al_JBC_SI [file mmc2.pdf]

Supporting Information

Binding mode between peptidyl-tRNA hydrolase and the peptidyl-A76 moiety of the substrate

Yuji Uehara [1], Ami Matsumoto [1], Tomonori Nakazawa [1], Akane Fukuta [2], Kaori Ando [2], Toshio Uchiumi [3], Natsuhisa Oka [2,4,5], Kosuke Ito [1,*]

1. Department of Biology, Faculty of Science, Niigata University, 8050, Ikarashi 2-no-cho, Nishi-ku, Niigata 950-2181, Japan.
2. Department of Chemistry and Biomolecular Science, Faculty of Engineering, Gifu University, 1-1 Yanagido, Gifu 501-1193, Japan.
3. The Institute of Science and Technology, Niigata University, 8050, Ikarashi 2-no-cho, Nishi-ku, Niigata 950-2181, Japan.
4. Institute for Glyco-core Research (iGCORE), Gifu University, 1-1 Yanagido, Gifu 501-1193, Japan.
5. Center for One Medicine Innovative Translational Research (COMIT), Gifu University, 1-1 Yanagido, Gifu 501-1193, Japan.

* Corresponding author: k-ito@bio.sc.niigata-u.ac.jp

This PDF file includes:
Supporting Experimental Procedures
Figures S1 to S7
Tables S1 to S3

Supporting Experimental Procedures

General information of the chemical synthesis of 3′-(N-acetyl-L-alanyl)amino-3′-deoxyadenosine

Commercially available reagents were used without purification. Dry organic solvents were prepared by appropriate procedures prior to use. Other organic solvents were reagent grade and used as received. All reactions in dry solvents were carried out under argon. Analytical thin-layer chromatography (TLC) was performed on Merck TLC plates (No. 5715) precoated with silica gel 60 F_{254}. Column chromatography was carried out using Kanto silica gel 60N (spherical, neutral, 63–210 μm), Fuji Sylisia Chromatorex NH-DM1020 (spherical, 100 μm), or Kanto silica gel 120 RP-18 (spherical, 40–50 μm). The ^{1}H and ^{13}C NMR spectra (400, 100 MHz) were recorded on a JNM-ECS-400 spectrometer (JEOL). Tetramethylsilane (TMS) (0.0 ppm) was used as the internal standard for ^{1}H NMR in $CDCl_3$. $CDCl_3$ (77.0 ppm) was used as the internal standard for ^{13}C NMR in $CDCl_3$. Sodium 3-trimethylsilyl-1-propanesulfonate was used as an external standard for ^{1}H and ^{13}C NMR in D_2O (0.0 ppm). ^{1}H NMR data are reported as follows: chemical shift (multiplicity, coupling constants, integration). Multiplicity is indicated as follows: s (singlet); d (doublet); dd (doublet of doublets); ddd (doublet of doublet of doublets); t (triplet); q (quartet); m (multiplet); br (broad). High resolution mass spectra were recorded on a Waters Xevo Q-Tof mass spectrometer (ESI-TOF).

Chemical synthesis of 3′-(N-acetyl-L-alanyl)amino-3′-deoxyadenosine

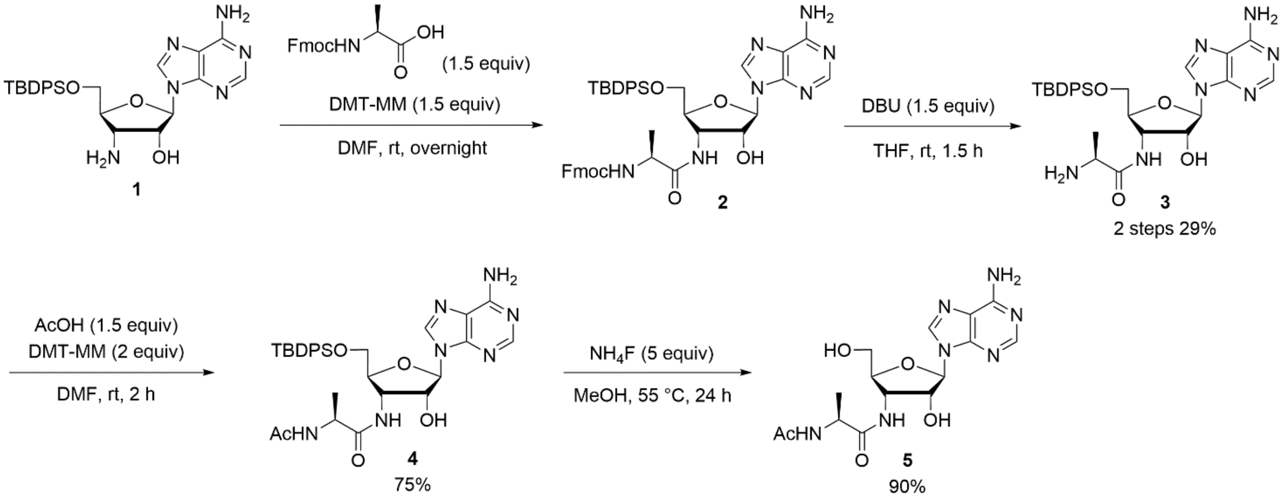

3′ -(L-Alanyl)amino-5′ -O-(tert-butyldiphenylsilyl)-3′ -deoxyadenosine 3

N-(9-Fluorenylmethoxycarbonyl)-L-alanine (0.582 g, 1.87 mmol) was dried by repeated co-evaporation with dry pyridine and dry toluene and dissolved in dry DMF (10.0 mL) under argon. 4-(4,6-Dimethoxy-1,3,5-triazin-2-yl)-4-methylmorpholinium chloride (DMT-MM) (0.517 g, 1.87 mmol) was added to the solution under stirring, and stirring was continued for 1 h at rt. 3′-Amino-5′-*O*-(*tert*-butyldiphenylsilyl)-3′-deoxyadenosine **1** (40) (0.628 g, 1.24 mmol) was placed in a separate flask, dried by repteated co-evaporation with dry pyridine and dry toluene, and dissolved in dry DMF (10.0 mL) under argon. The resulting solution of **1** was added dropwise to the solution containing the alanine derivative and stirring was continued overnight at rt. The solution was diluted with AcOEt (70 mL) and washed with H_2O (70 mL). The aqueous layer was separated and extracted with AcOEt (4 × 40 mL). The organic layers were combined, dried over Na_2SO_4, filtered, and concentrated under reduced pressure. The residue was purified by column chromatography on silica gel (Kanto silica gel 60N, CH_2Cl_2–MeOH = 90:10). The fractions containing compound **2** were collected and concentrated under reduced pressure. The residue (0.332 g) was dried by repeated co-evaporation with dry MeCN and dissolved in dry THF (4.0 mL) under argon. Dry 1,8-diazabicyclo[5.4.0]-undec-7-ene (DBU) (0.092 mL, 0.62 mmol) was added dropwise to the solution under stirring, and stirring was continued for 1.5 h at rt. Then, the solution was diluted with AcOEt (30 mL) and washed with a saturated aqueous solution of $NaHCO_3$ (15 mL). The aqueous layer was separated and extracted with AcOEt (2 × 15 mL). The organic layers were combined, dried over Na_2SO_4, filtered, and concentrated under reduced pressure. Finally, the residue was purified by column chromatography on silica gel (Fuji Sylisia Chromatorex NH-DM1020, CH_2Cl_2–MeOH = 100:0→96:4) to afford **3** (0.206 g, 0.358 mmol, 29%) as a pale yellow solid. ^1H NMR (400 MHz, $CDCl_3$) δ 8.19 (s, 1H), 8.18 (s, 1H), 7.76 (d, *J* = 7.8 Hz, 1H), 7.67–7.61 (m, 4H), 7.40–7.30 (m, 6H), 6.77 (br, 1H), 6.11 (s, 1H), 4.78–4.71 (m, 1H), 4.69–4.66 (m, 1H), 4.28–4.24 (m, 1H), 4.05 (d, *J* = 11.8 Hz, 1H), 3.85 (d, *J* = 11.8 Hz, 1H), 3.49 (q, *J* = 6.8 Hz, 1H), 1.27 (d, *J* = 6.8 Hz, 3H), 1.04 (s, 9H). ^{13}C NMR (100 MHz, $CDCl_3$) δ 176.2, 155.7, 152.5, 148.6, 138.4, 135.7, 135.4, 132.9, 132.6, 129.8, 129.7, 127.7, 127.7, 119.8, 90.8, 83.8, 74.4, 63.3, 50.7, 50.0, 26.8, 21.6, 19.1. HRMS (ESI-TOF) *m*/*z*: $[M + H]^+$ calcd for $C_{29}H_{38}N_7O_4Si^+$ 576.2749; found 576.2728.

3′ -(N-Acetyl-L-alanyl)amino-5′ -O-(tert-butyldiphenylsilyl)-3′ -deoxyadenosine 4

DMT-MM (0.192 g, 0.694 mmol) was added to a solution of acetic acid (0.029 mL, 0.51

mmol) in dry DMF (0.50 mL) under stirring and stirring was continued under argon at rt for 30 min. Compound **3** (0.195 g, 0.339 mmol) was dried by repeated co-evaporation with dry pyridine and dry toluene and dissolved in dry DMF (2.5 mL) in a separate flask under argon. The second solution containing **3** was added dropwise to the first solution and stirred for 2 h. The solution was diluted with AcOEt (20 mL) and washed with H_2O (50 mL). The aqueous layer was separated and extracted with AcOEt (3 × 40 mL). The organic layers were collected, dried over Na_2SO_4, filtered, and concentrated under reduced pressure. Finally, the residue was purified by column chromatography on silica gel (Fuji Sylisia Chromatorex NH-DM1020, CH_2Cl_2–MeOH = 100:0→96:4) to afford **4** (0.157 g, 0.254 mmol, 75%) as a pale yellow solid. ^1H NMR (400 MHz, $CDCl_3$) δ 8.26 (s, 1H), 8.12 (s, 1H), 7.93 (br, 1H), 7.72–7.55 (m, 4H), 7.39–7.22 (m, 6H), 6.05 (s, 1H), 5.03–4.83 (m, 2H), 4.73–4.62 (m, 1H), 4.23–4.09 (m, 1H), 4.04–3.94 (m, 1H), 3.79–3.65 (m, 1H), 1.93 (s, 3H), 1.34 (s, 3H), 0.99 (s, 9H). ^{13}C NMR (100 MHz, $CDCl_3$) δ 174.0, 171.0, 155.5, 152.4, 148.4, 138.5, 135.6, 135.4, 132.8, 132.6, 129.7, 127.8, 127.7, 119.5, 90.9, 82.9, 74.1, 62.8, 49.8, 48.9, 26.8, 22.9, 19.0. HRMS (ESI-TOF) *m/z*: $[M + H]^+$ calcd for $C_{31}H_{40}N_7O_5Si^+$ 618.2855; found 618.2862.

3′ -(N-Acetyl-L-alanyl)amino-3′ -deoxyadenosine 5

NH_4F (0.021 g, 0.57 mmol) was added to a solution of **4** (0.0656 g, 0.106 mmol) in dry MeOH (3.0 mL) under argon. The solution was stirred at 55 °C for 24 h and concentrated under reduced pressure. The residue was then purified by reversed phase column chromatography (Kanto silica gel 120 RP-18, H_2O–MeCN = 100:0→80:20) to afford **5** (0.0364 g, 0.0959 mmol, 90%) as a white solid. ^1H NMR (400 MHz, D_2O) δ 8.18 (s, 1H), 7.91 (s, 1H), 5.94 (d, J = 2.2 Hz, 1H), 4.63–4.57 (m, 2H), 4.30 (q, J = 7.2 Hz, 1H), 4.21 (ddd, J = 7.8, 3.8, 2.4 Hz, 1H), 3.90 (dd, J = 13.0, 2.4 Hz, 1H), 3.70 (dd, J = 13.0, 3.8 Hz, 1H), 1.98 (s, 3H), 1.36 (d, J = 7.2 Hz, 3H). ^{13}C NMR (100 MHz, D_2O) δ 176.3, 174.7, 155.5, 152.8, 148.3, 140.2, 118.9, 90.2, 83.1, 74.2, 61.0, 51.2, 50.5, 22.2, 17.5. HRMS (ESI-TOF) *m/z*: $[M + H]^+$ calcd for $C_{15}H_{22}N_7O_5^+$ 380.1677; found 380.1687.

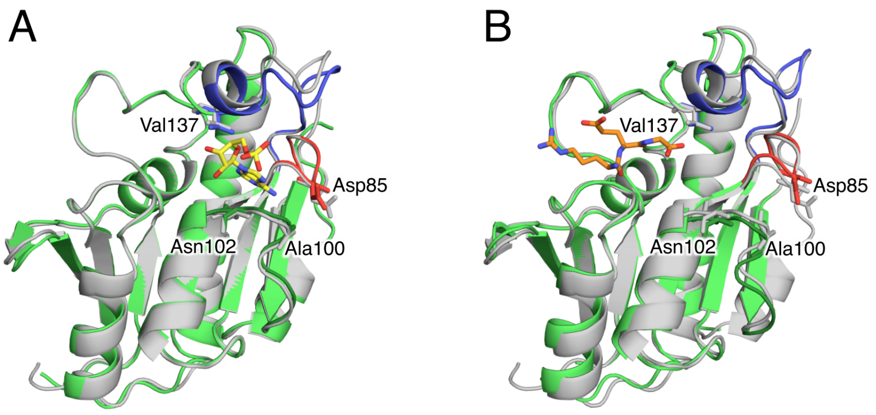

Figure S1. Superimpositions of *Tt*Pth structures. (**A**) Superimposition of the structures of apo *Tt*Pth (gray) (PDB ID: 5ZX8) (35) and *Tt*Pth•AMP (green). (**B**) Superimposition of the structures of apo *Tt*Pth (gray) (PDB ID: 5ZX8) (35) and *Tt*Pth•tripeptide (green). The base loops, gate loops, and lid loops of *Tt*Pth•AMP and *Tt*Pth•tripeptide are colored dark green, red, and blue, respectively. Asp85, Ala100, Asn102, and Val137 are shown as stick models. AMP and tripeptide are shown as yellow and orange stick models, respectively.

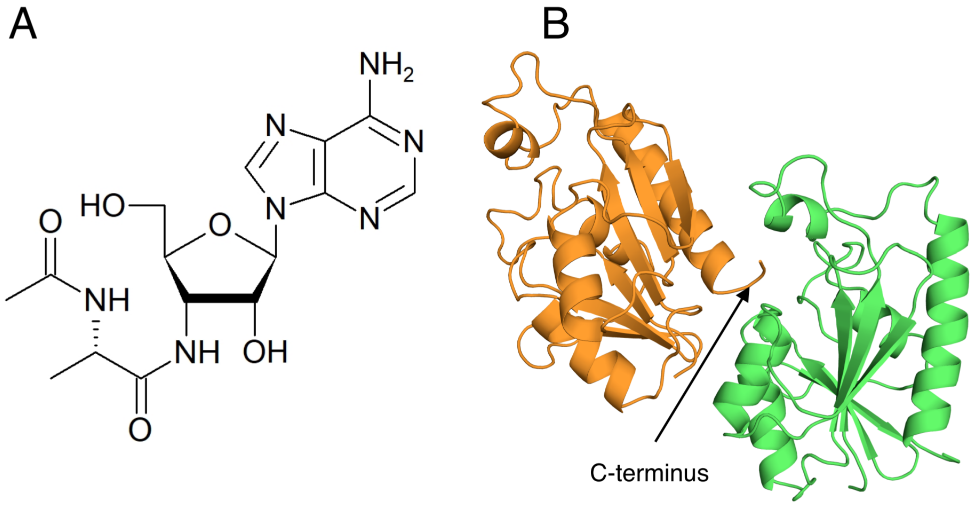

Figure S2. Formation of the *Tt*Pth•tripeptide crystal. (**A**) Chemical structure of 3′-(*N*-acetyl-L-alanyl)amino-3′-deoxyadenosine. (**B**) Packing of *Tt*Pth molecules in the crystal. *Tt*Pth molecules are shown by cartoon models, with a reference molecule in green and a neighboring molecule in orange.

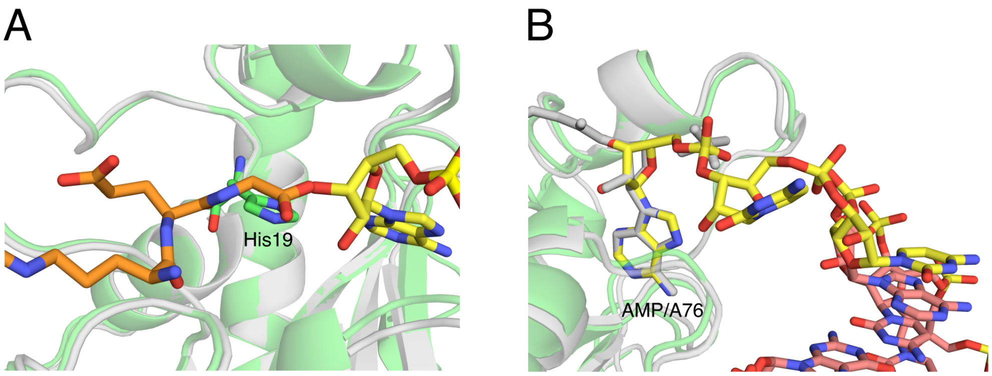

Figure S3. Superimpositions of complex structures of *Tt*Pth. (**A**) Superimposition of the structures of *Tt*Pth•AMP and *Tt*Pth•tripeptide. The structures of *Tt*Pths in *Tt*Pth•AMP and *Tt*Pth•tripeptide are shown as green and gray cartoon models, respectively. AMP and the tripeptide are shown as yellow and orange stick models, respectively. (**B**) Superimposition of the structures of *Tt*Pth•AMP and the *Ec*Pth•CCA-acceptor-TΨC domain of tRNA (PDB ID: 3VJR) (27). *Tt*Pth and AMP are shown as a gray cartoon model and a gray stick model, respectively. *Ec*Pth is shown as a green cartoon model. The CCA terminus and the acceptor stem of tRNA are shown as yellow and pink stick models, respectively. The structure of the CCA terminus is modified to superimpose the A76 moiety on AMP. In both panels (A) and (B), to make the stick models more visible, the cartoon models are shown as semi-transparent.

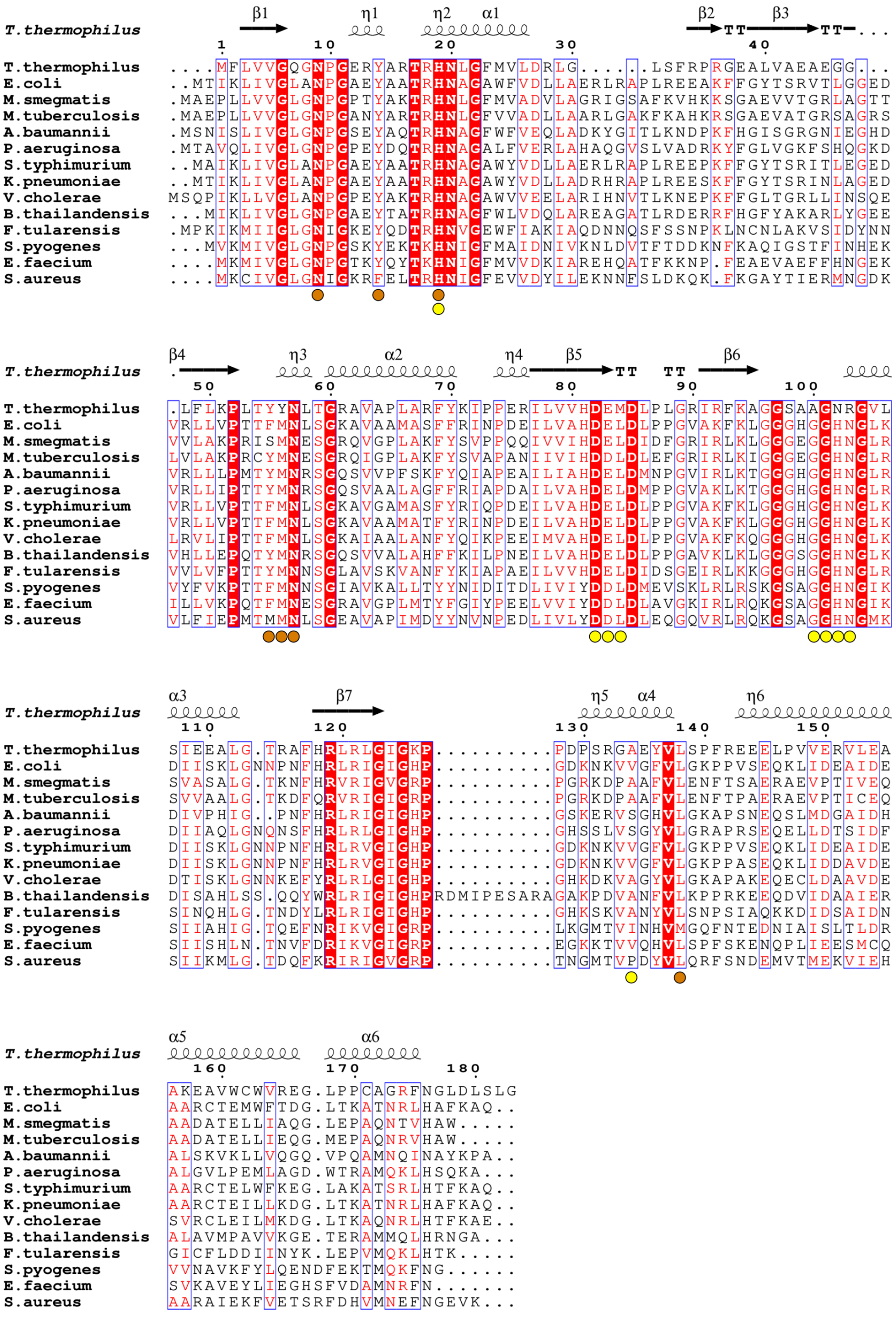

Figure S4. Amino acid sequence alignment of Pths. The amino acid sequence of *Tt*Pth was aligned with those of the structure-determined Pths. Conserved amino acid residues are

shown by red characters. Strictly conserved amino acid residues are indicated by a red background. The secondary structure of *Tt*Pth is shown above the sequence (α = α-helix, β = β-strand, η = 3_{10}-helix, TT = β-turn). The yellow and orange dots below the sequences indicate the residues that participate in the interactions with AMP and the tripeptide, respectively.

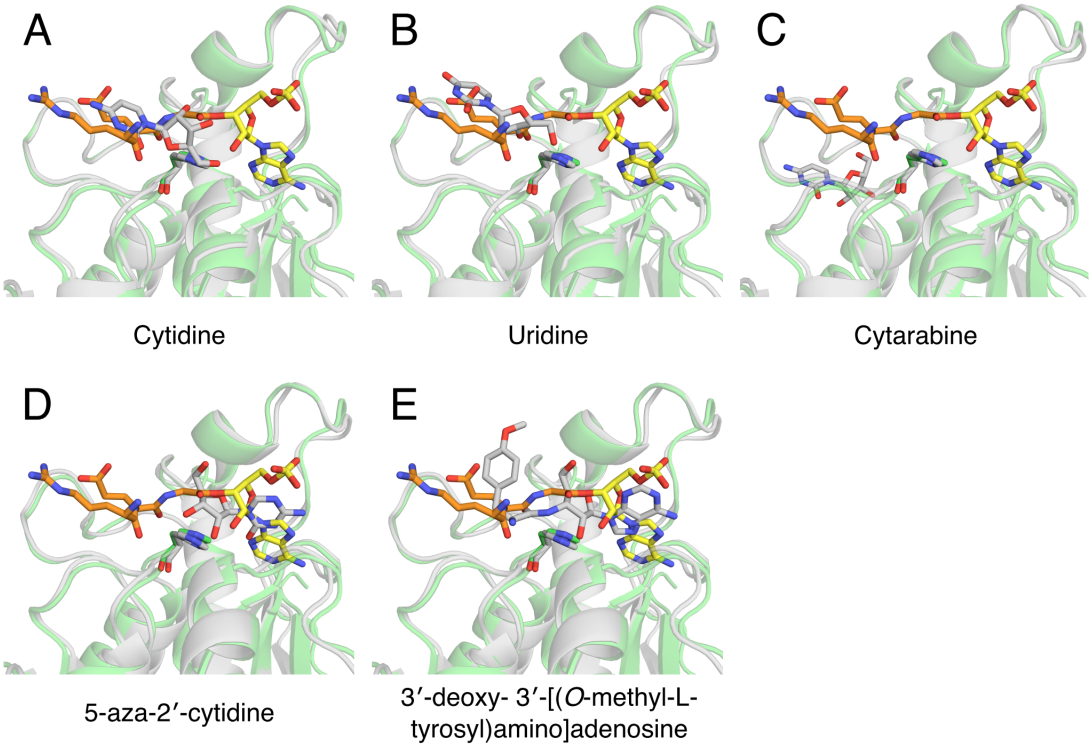

Figure S5. Comparisons of binding modes of small inhibitor compounds and peptidyl-A76 to Pths. Superimpositions of the *Tt*Pth•peptidyl-A76 complex model and (**A**) *Acinetobacter baumannii* Pth (*Ab*Pth)•cytidine complex (PDB ID: 4JWK) (29), (**B**) *Ab*Pth•uridine complex (PDB ID: 4JX9) (29), (**C**) *Ab*Pth•cytarabine complex (PDB ID: 5Y9A) (31), (**D**) *Pseudomonas aeruginosa* Pth (*Pa*Pth)•5-aza-2′-cytidine complex (PDB ID: 4QD3) (30), and (**E**) *Pa*Pth•3′-deoxy-3′-[(*O*-methyl-L-tyrosyl)amino]adenosine complex (PDB ID: 4QBK) (30). *Tt*Pths are shown as cartoon models. Peptidyl-A76 and inhibitor compounds are shown as stick models. The color coding of the *Tt*Pth•peptidyl-A76 complex model is the same as in Fig. 3A. Pth•inhibitor compound complexes are shown in gray. In all panels (A) to (E), to make the stick models more visible, the cartoon models are shown as semi-transparent.

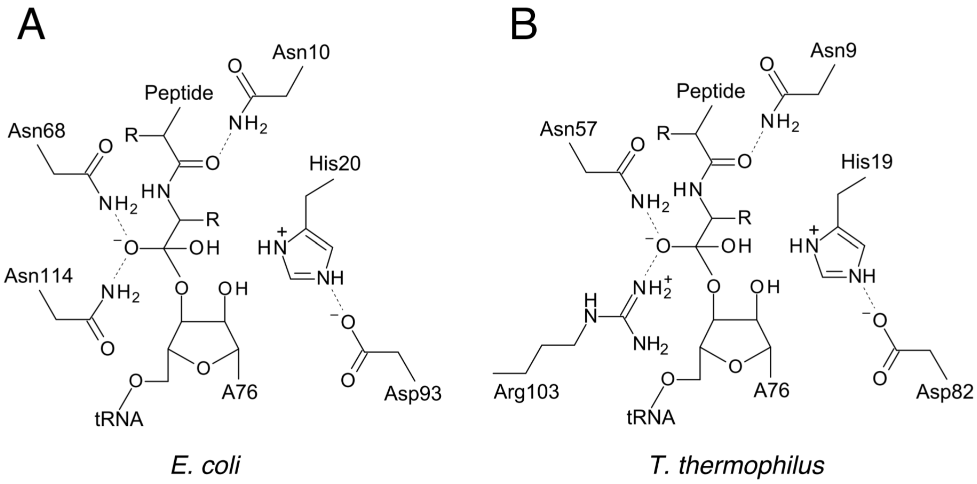

Figure S6. Comparison of the tetrahedral intermediates between *E. coli* Pth and *T. thermophilus* Pth. Models of the tetrahedral intermediates of (**A**) *E. coli* Pth and (**B**) *T. thermophilus* Pth.

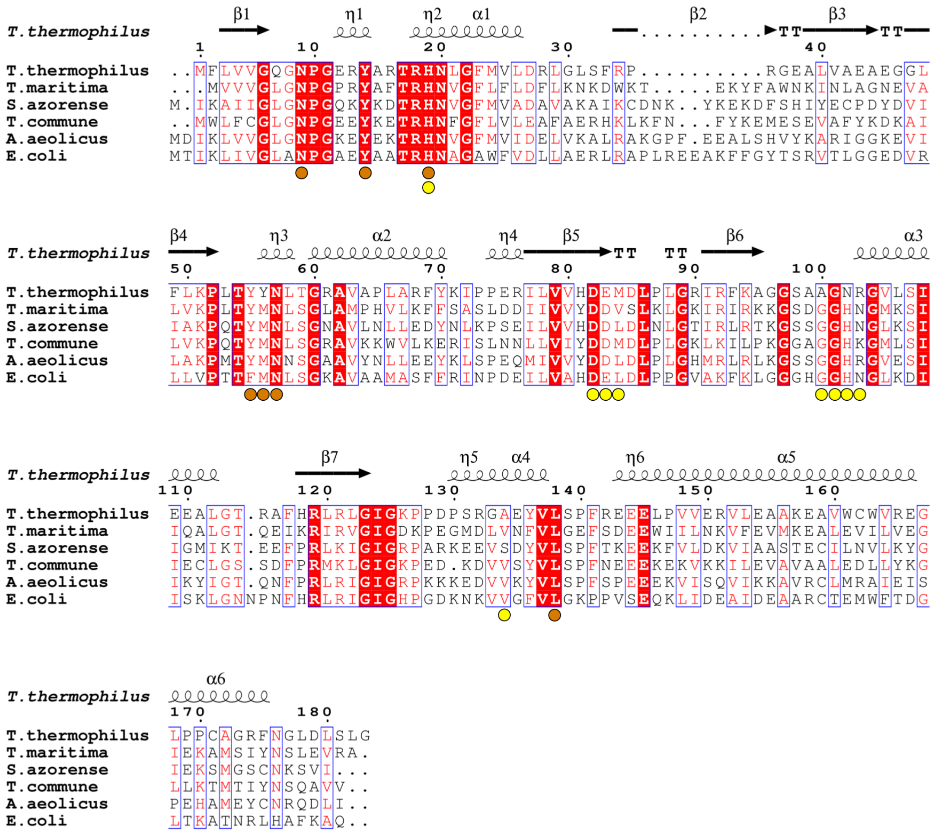

Figure S7. Amino acid sequence alignment of Pths from thermophilic bacteria. The description of the figure is the same as that for Fig. S4. For reference, the amino acid sequence of *E. coli* Pth is also shown as a representative Pth from mesophilic bacteria.

Table S1. List of the interactions between *Tt*Pth and AMP.

Hydrogen bonds

*Tt*Pth		AMP	distance (Å)
Glu83	OE2	N6	2.78
Met84	N	N1	2.99
Ala100	O	N6	2.88
Arg103	N	O2'	3.27

van der Waals contacts

*Tt*Pth		AMP	distance (Å)
His19	CE1	N3	3.35
Asp82	O	C2	3.65
Glu83	CA	N1	3.71
Glu83	CD	N6	3.89
Glu83	OE2	C6	3.63
Glu83	C	N1	3.87
Met84	N	C2	3.73
Met84	N	C6	3.68
Met84	CB	N1	3.72
Met84	CB	C6	3.80
Met84	CG	N1	3.90
Met84	CG	C2	3.85
Met84	CE	O4'	3.45
Met84	CE	N3	3.87
Met84	CE	C4	3.37
Met84	CE	C5	3.59
Met84	CE	N7	3.74
Met84	CE	C8	3.54
Met84	CE	N9	3.45
Ala100	CB	N6	3.90
Ala100	C	N6	3.77
Ala100	O	C5	3.83
Ala100	O	C6	3.68
Gly101	CA	N7	3.65
Gly101	C	C5	3.78
Gly101	C	N7	3.18
Gly101	C	C8	3.49
Gly101	O	C2'	3.84
Gly101	O	C8	3.12
Asn102	N	C5	3.61
Asn102	CA	C4	3.80
Asn102	CA	C5	3.65
Asn102	CB	N1	3.84
Asn102	CB	C6	3.75
Asn102	ND2	C2	3.29
Arg103	CA	O2'	3.89
Arg103	CB	O2'	3.37
Arg103	CG	O2'	3.35
Arg103	CD	O2'	3.74
Ala134	N	C4'	3.81
Ala134	N	C5'	3.80

Table S2A. List of the interactions between *Tt*Pth and tripeptide.

Hydrogen bonds

tripeptide		*Tt*Pth		distance (Å)
Arg165	O	Asn57	N	2.96
Glu166	O	Asn9	ND2	3.03
Gly167	O	Asn57	ND2	2.90

van der Waals contacts

tripeptide		*Tt*Pth		distance (Å)
Arg165	CB	Tyr55	CB	3.75
Arg165	CB	Tyr55	CG	3.73
Arg165	CG	Tyr55	CG	3.69
Arg165	CG	Tyr55	CD2	3.50
Arg165	CD	Tyr55	CE2	3.55
Arg165	CD	Tyr55	CD2	3.57
Arg165	NE	Tyr55	CE2	3.89
Arg165	C	Tyr55	CB	3.81
Arg165	C	Asn57	ND2	3.60
Arg165	O	Tyr55	CB	3.52
Arg165	O	Tyr55	C	3.84
Arg165	O	Tyr56	CA	3.88
Arg165	O	Tyr56	C	3.89
Arg165	O	Asn57	CA	3.72
Arg165	O	Asn57	CB	3.34
Arg165	O	Asn57	CG	3.26
Glu166	CA	Asn9	CG	3.84
Glu166	CA	Asn9	OD1	3.34
Glu166	CA	Asn9	ND2	3.70
Glu166	CB	Asn9	CG	3.67
Glu166	CB	Asn9	OD1	3.48
Glu166	CB	Asn9	ND2	3.59
Glu166	CB	Tyr14	OH	3.87
Glu166	CG	Asn9	OD1	3.56
Glu166	CG	Tyr14	CZ	3.61
Glu166	CG	Tyr14	OH	3.21
Glu166	CG	Tyr55	CD1	3.35
Glu166	CG	Tyr55	CE1	3.67
Glu166	CD	Tyr14	OH	3.17
Glu166	OE2	Tyr55	CD1	3.73
Glu166	OE2	Tyr55	CE1	3.30
Glu166	OE2	Tyr55	CZ	3.79
Glu166	C	Asn9	ND2	3.80
Glu166	O	His19	CD2	3.27
Glu166	O	Tyr56	CB	3.78
Glu166	O	Leu138	CD2	3.53
Gly167	C	Asn57	ND2	3.64

The interactions are arranged by amino acid number of the tripeptide (neighboring molecule).

Table S2B. List of the interactions between *Tt*Pth and tripeptide.

Hydrogen bonds

*Tt*Pth		tripeptide		distance (Å)
Asn9	ND2	Glu166	O	3.03
Asn57	N	Arg165	O	2.96
Asn57	ND2	Gly167	O	2.90

van der Waals contacts

*Tt*Pth		tripeptide		distance (Å)
Asn9	CG	Glu166	CA	3.84
Asn9	CG	Glu166	CB	3.67
Asn9	OD1	Glu166	CA	3.34
Asn9	OD1	Glu166	CB	3.48
Asn9	OD1	Glu166	CG	3.56
Asn9	ND2	Glu166	CA	3.70
Asn9	ND2	Glu166	C	3.80
Asn9	ND2	Glu166	CB	3.59
Tyr14	CZ	Glu166	CG	3.61
Tyr14	OH	Glu166	CB	3.87
Tyr14	OH	Glu166	CG	3.21
Tyr14	OH	Glu166	CD	3.17
His19	CD2	Glu166	O	3.27
Tyr55	CB	Arg165	CB	3.75
Tyr55	CB	Arg165	C	3.81
Tyr55	CB	Arg165	O	3.52
Tyr55	CG	Arg165	CB	3.73
Tyr55	CG	Arg165	CG	3.69
Tyr55	CD1	Glu166	CG	3.35
Tyr55	CD1	Glu166	OE2	3.73
Tyr55	CE1	Glu166	CG	3.67
Tyr55	CE1	Glu166	OE2	3.30
Tyr55	CZ	Glu166	OE2	3.79
Tyr55	CE2	Arg165	CD	3.55
Tyr55	CE2	Arg165	NE	3.89
Tyr55	CD2	Arg165	CG	3.50
Tyr55	CD2	Arg165	CD	3.57
Tyr55	C	Arg165	O	3.84
Tyr56	CA	Arg165	O	3.88
Tyr56	CB	Glu166	O	3.78
Tyr56	C	Arg165	O	3.89
Asn57	CA	Arg165	O	3.72
Asn57	CB	Arg165	O	3.34
Asn57	CG	Arg165	O	3.26
Asn57	ND2	Arg165	C	3.60
Asn57	ND2	Gly167	C	3.64
Leu138	CD2	Glu166	O	3.53

The interactions are arranged by amino acid number of the reference *Tt*Pth molecule.

Table S3. Sequences of primers used in this study.

No.	Name	Sequence (5' to 3')
1	ΔC16 sense	GGTGCGGGAGGGTTAACCCCCCTGCGCC
2	ΔC16 anti-sense	GGCGCAGGGGGGTTAACCCTCCCGCACC
3	E83A sense	CACGACGCGATGGACCTCCCTTTG
4	E83A anti-sense	GAGGTCCATCGCGTCGTCGACCAC
5	N102A sense	GCGCCGCCGGGGCGCGGGGCGTGCTTTC
6	N102A anti-sense	CACGCCCCGCGCCCCGGCGGCGCTC
7	R103A sense	GCCGGGAACGCGGGCGTGCTTTC
8	R103A anti-sense	CACGCCCGCGTTCCCGGCGGC
9	L138A sense	CGGAGTACGTGGCCTCCCCCTTCCG
10	L138A anti-sense	CGGAAGGGGGAGGCCACGTACTCCG
11	T7-tRNA^{Lys} forward	GAAATTAATACGACTCACTATAGGGTCGTTAGCTCAGTTGG
12	T7-tRNA^{Lys} reverse	TGm*GTGGGTCGTGCAGGATTCG

*Gm: 2'-O-methyl guanosine
